# Two alternative splicing variants of a wheat gene *TaNAK1*, *TaNAK1.1* and *TaNAK1.2*, differentially regulate flowering time and plant architecture leading to differences in seed yield of transgenic *Arabidopsis*

**DOI:** 10.3389/fpls.2022.1014176

**Published:** 2022-12-01

**Authors:** Baowei Wu, Xiaoyu Zhang, Kunzhi Hu, Haoyuan Zheng, Siyu Zhang, Xiangli Liu, Meng Ma, Huixian Zhao

**Affiliations:** College of Life Sciences, Northwest A & F University, Yangling, Shaanxi, China

**Keywords:** wheat, TaNAK1, alternative splicing, flowering time, plant architecture, seed yield

## Abstract

In wheat production, appropriate flowering time and ideal plant architecture are the prerequisites for high grain yield. Alternative splicing (AS) is a vital process that regulates gene expression at the post-transcriptional level, and AS events in wheat have been found to be closely related to grain-related traits and abiotic stress tolerance. However, AS events and their biological roles in regulating flowering time and plant architecture in wheat remain unclear. In this study, we report that *TaNAK1* undergoes AS, producing three splicing variants. Molecular characterization of *TaNAK1* and its splicing variants demonstrated that all three protein isoforms have a conserved NB-ARC domain and a protein kinase domain, but the positions of these two domains and the length of the protein kinase domains are different among them, implying that they may have different three-dimensional structures and therefore have different functions. Further investigations showed that the two splicing variants of *TaNAK1, TaNAK1.1* and *TaNAK1.2*, exhibited different expression patterns during wheat growth and development, while the other one, *TaNAK1.3*, was not detected. Subcellular localization demonstrated that TaNAK1.1 was mainly localized in the cytoplasm, while TaNAK1.2 was localized in the nucleus and cytoplasm. Both TaNAK1.1 and TaNAK1.2 exhibit protein kinase activity *in vitro*. Ectopic expression of *TaNAK1.1* and *TaNAK1.2* in *Arabidopsis* demonstrated that these two splicing variants play opposite roles in regulating flowering time and plant architecture, resulting in different seed yields. *TaNAK1.2* positive regulates the transition from vegetative to reproductive growth, plant height, branching number, seed size, and seed yield of *Arabidopsis*, while *TaNAK1.1* negatively regulates these traits. Our findings provide new gene resource for regulating flowering time and plant architecture in crop breeding for high grain yield.

## Introduction

Wheat (*Triticum aestivum* L.), one of the major staple crops worldwide, provides more than one fifth calories and protein for mankind. Improving wheat production is critical to world food and nutrition security (FAO, http://faostat.fao.org). In crop production, appropriate flowering and ideal plant architecture are the prerequisites for high yield (Wang et al., 2018). The appropriate flowering time is crucial for wheat to deal with adverse environmental factors, such as cold, freezing, high temperature, rainstorm, and various diseases, to complete life generation and to maintain species reproduction (Worland, 1996). Meanwhile, it is also conducive to get high yield and better quality through the coordination of vegetative and reproductive growth (Kamran et al., 2014). Therefore, in the long-term practice of wheat cultivation, domestication and production, flowering time has been regarded as one of the important agronomic traits. Plant architecture refers to the structure and three-dimensional organization of all organs of a plant, which includes aspects such as stem height, tillering/branching pattern and number, leaf shape and arrangement, the number of inflorescences/flowers and their distribution, as well as root morphology and distribution. The significance of plant architecture in agriculture is that it is regulated by both genetic and environmental factors. This confers extensive phenotypic plasticity that enables plants to adapt to different environment, facilitating selection for favorable traits such as grain yield and harvest indices (Reinhardt and Kuhlemeier, 2002). Crop plant architecture is regarded as one of the most crucial factors that affect grain yield. Wheat breeders have been focused on its architecture, including plant height, tiller number and angle, leaf shape, size, and angle, and spike morphology since the Green Revolution, the development and cultivation of semi-dwarf wheat and rice varieties that have greatly increased crop production since 1960 (Wang et al., 2018; Li et al., 2019). Therefore, it is of great significance to explore and identify the genes regulating flowering time and plant architecture in wheat, understand their molecular mechanism, and realize the fine regulation of flowering time and plant architecture, so as to improve grain yield and ensure food security.

Alternative splicing (AS) is a vital process for gene expression regulation at the post-transcriptional level, in which a pre-mRNA can produce multiple transcripts through different splicing modes to improve transcriptome plasticity and proteome diversity, to increase functional complexity (Yu et al., 2016; Ananda et al., 2022), and even to engender trait diversity (Gao et al., 2021). Therefore, AS plays an important role in plant adaptation and evolution. AS mainly includes exon skipping (ES), intron retention (IR), alternative 5’ splice site (alt 5’SS), alternative 3’ splice site (alt 3’SS), variable first exon (VFE), variable last exon (VLE) and mutually exclusive exons (MEE) (Ule and Blencowe, 2019). AS can generate truncated proteins, disrupting main domains to form non-functional proteins (Kalyna et al., 2012) or annulling interaction with other proteins and thereby impeding the formation of functional protein complexes (Qin et al., 2017). Moreover, some splicing variants can antagonistically compete with other variant and interfere with its function in a negative manner (Posé et al., 2013; Ren et al., 2021). Increasing evidences have demonstrated that AS participates in the regulation of plant growth and development. In *Arabidopsis*, the MADS-box transcription factor genes *FLOWERING LOCUS M* (*FLM*) and *SHORT VEGETATIVE PHASE* (*SVP*) have key functions in regulation of flowering. *FLM* undergoes temperature-dependent alternative splicing, and antagonistic *FLM* variants, *FLM*-β and *FLM*-δ, compete for interaction with the floral repressor *SVP* to regulate flowering (Posé et al., 2013). AS of *Delay of Germination1 (DOG1)* produces two mRNA variants, *lgDOG1* and *shDOG1*. In contrast to *lgDOG1, shDOG1* is translated and functions to promote seed dormancy in *Arabidopsis* (Cyrek et al., 2016). AS is involved in the regulation of some grain-related traits. In rice, AS of *OsLG3b*, a gene encoding *MADS-box transcription factor1* (*OsMADS1*), produces the truncated proteins that are positively associated with grain length and contributes to grain yield (Yu et al., 2018). In *Oryza officinalis Wall. ex Watt, MKK3* encoding mitogen-activated protein kinase 3 is subject to alternative splicing and produces splicing variants, and overexpressing four of the five OsMKK3 splicing variants leads to reduced grain length and width (Pan et al., 2021). Wheat has very huge and complex genome, and substantial wheat genes have complicated roles (Singh et al., 2018). The genome-wide AS profiling uncovered a very complex AS landscape in wheat, exhibiting that almost 22% of genes occur AS (Yu et al., 2020). Increasing evidences have uncovered that AS events in wheat are enormously related to grain-related traits such as polyphenol oxidase activity (Sun et al., 2010), starch synthase activity (Zhou et al., 2018), and grain weight and size (Ren et al., 2021). AS is also related to abiotic stresses tolerance (Egawa et al., 2006; Liu et al., 2018) and disease resistance (Wang et al., 2020; Sánchez-Martín et al., 2021). However, AS events and their biological roles in regulating wheat flowering time and plant architecture are poorly understood.

Our previous study identified a wheat specific-miRNA tae-miR5048 from wheat small RNA (sRNA) library, which is highly expressed in seedlings and flag leaves (Han et al., 2014). Recently, a RLK gene (ID TraeCS4B01G313900) in IWGSC RefSeq v1.0) (https://urgi.versailles.inra.fr/download/iwgsc/IWGSC_RefSeq_Annotations/v1.0/), was identified to be one of tae-miR5048 target genes by using psRNA Target software (http://plantgrn.noble.org/psRNATarget/) and Degradome sequencing (unpublished). However, its biological function remains elusive. In this study, we found that this RLK gene is subject to AS and produces three splicing variants encoding the NB-ARC (Nucleotide-binding adaptor shared by APAF-1, R proteins and CED-4) domain and protein kinase domain proteins, thereby named as *TaNAK1.* Molecular characterization of *TaNAK1* and its three splice variants demonstrated that all three protein isoforms have a conserved NB-ARC domain and a protein kinase domain, but the positions of these two domains and the length of the protein kinase domains are different among them, implying that they may have different three-dimensional structures and therefore have different biological functions. Further investigations showed that the two splicing variants of *TaNAK1, TaNAK1.1* and *TaNAK1.2*, exhibited different expression patterns during wheat growth and development, while the other splice variant, *TaNAK1.3*, was not detected. Subcellular localization showed that TaNAK1.1 was mainly localized in the cytoplasm, while TaNAK1.2 was localized in the nucleus and cytoplasm. Both TaNAK1.1 and TaNAK1.2 exhibit the activity of protein kinase *in vitro*. Ectopic expression of *TaNAK1.1* and *TaNAK1.2* in *Arabidopsis* demonstrated that these two splicing variants play opposing roles in regulating flowering time and plant architecture, resulting in different seed yields. Our findings provide new gene resource for regulating flowering time and plant architecture in crop breeding for high yield.

## Materials and methods

### Plant materials and growth conditions

Wheat cultivar cv. Chinese Spring was used to clone the cDNA of *TaNAK1*, and winter wheat cv. Xiaoyan 6 was used to determine the expression patterns of the splicing variants of *TaNAK1* during wheat growth and development. After imbibition, the seeds of Chinese Spring were placed on wet filter paper and germinated at the condition of 22°C, 16 h light/8 h dark, and 75% relative humidity. Leaf and root samples were collected from wheat seedlings at the two-leaf stage, rapidly frozen in liquid nitrogen, and then stored at -80°C for RNA extraction and cDNA cloning. Xiaoyan 6 was planted in the experimental plots at Northwest A & F University, Yangling, Shaanxi, China (longitude 108°4′E, latitude 34°17′N) during the natural growing seasons in 2018 and 2020. Ten wheat organs/tissues, including root (R), stem (S), leaf (L) of five-leaf-stage seedlings, flag leaf (FL) from wheat plants at heading stages, the 5 cm long young ear (YS5) from wheat plants at booting stage, and the 10 cm long spike (YS10) from wheat plants at heading stage, the grains 5, 10, 15, 20, and 25 days post-anthesis (GR5, GR10, GR15, and GR20, respectively), were collected and rapidly frozen in liquid nitrogen and stored at -80°C for further usage, three independent biological replicates being included for individual tissues/organs.

*Arabidopsis* (ecotype Columbia-0) was used to conducted genetic transformation in this study. The seeds of *Arabidopsis* were sterilized and sowed after 2 days imbibition at 4°C, and the plants were cultured in a phytotron with 22°C, the photoperiod of 16 h light/8 h dark, the relative humidity of 70% and light intensity of 130-150 μmol/m^2^/s.

### Bioinformatics analysis of *TaRK4B-1* in wheat

A wheat gene (ID TraeCS4B01G313900), which was identified as one of the tae-miR5048 targets (unpublished data), is annotated as a receptor kinase gene (RK) and is subject to AS, generating three splicing variants (TraeCS4B01G313900.1, TraeCS4B01G313900.2, and TraeCS4B01G313900.3), based on the annotation in IWGSC RefSeq v1.0 (https://urgi.versailles.inra.fr/download/iwgsc/IWGSC_RefSeq_Annotations/v1.0/). Here, the gene is tentatively named as *TaRK4B-1*, and the three splicing variants are named as *TaRK4B-1.1*, *TaRK4B-1.2*, and *TaRK4B-1.3*, respectively. The genome DNA, cDNA, and the CDS sequences of *TaRK4B-1* were downloaded from IWGSC RefSeq v1.0, and used to analyze the gene structure. SMART (http://smart.embl.de/) was used to identify conserved domains contained in target proteins. DNAMAN v9 (https://www.lynnon.com/qa.html) was used for multi-sequences alignment.

### RNA extraction and cDNA synthesis

Total RNA of different organs/tissues from wheat or *Arabidopsis* was extracted using Polysaccharide and Polyphenol total RNA isolation Kit (BioTeke Corporation, Beijing, China) according to the manufacturer’s instruction. The quality of RNA samples obtained was assessed by 2.0% agarose gel electrophoresis. First strand cDNA synthesis was performed using Takara’s reverse transcription kit PrimeScript ™ IV 1st strand cDNA Synthesis Mix (Takara, Japan).

### Semi-quantitative reverse transcriptase-polymerase chain reaction

Because of the very high sequence similarity of *TaNAK1* splicing variants, it was not possible to design specific primers for quantitative analysis of individual splicing variants through real-time quantitative RT-PCR (qRT-PCR). Therefore, the expression levels of alternative splicing variants of *TaNAK1* in different developmental organs/tissues of wheat cv. Xiaoyan 6 were detected by semi-qRT-PCR with gene-specific primer pair (**Supplementary Table S1**) and the cDNA from different wheat tissues as templates, *TaACTIN* (TraesCS1A01G020500) being used as a reference gene.

### cDNA cloning of *TaNAK1* gene in wheat

Due to the very high sequence similarity of *TaNAK1* splicing variants, a universal primer pair (*TaNAK1*-F/R) that can amplify all the alternative splicing variants of *TaNAK1* was designed (**Supplementary Table S1**) according to the CDS sequences of *TaNAK1* downloaded from IWGSC RefSeq v1.0. The alternative splicing variants of *TaNAK1* were amplified by PCR with the primer pair and wheat leaf and grain cDNA mixture as template. PCR products were purified and ligated to a pMD™19-T vector (Takara Biotechnology (Dalian) Co. Ltd., Japan) for sequencing. At least five clones of each PCR product were selected for sequencing.

### Subcellular localization of TaNAK1.1 and TaNAK1.2

In order to reveal the subcellular localization of TaNAK1.1 and TaNAK1.2, the coding sequences of *TaNAK1.1* and *TaNAK1.2* were separately amplified using the primer pair containing restriction enzyme sites TaNAK1-SalI-F/TaNAK1-BamHI-R (**Supplementary Table S1**) and pMD19-T-*TaNAK1.1* and pMD19-T-*TaNAK1.2* as a template, respectively. The coding sequences of the amino terminus of two protein isoforms (1-257aa and 1-353 aa in TaNAK1.1, 1-306aa and 1-421 aa in TaNAK1.2) that include only their kinase domains or both the kinase domains and the NA-ARC domains were also separately amplified with primer pairs TaNAK1-HindIII-F/TaNAK1.1-N1-SalI-R, TaNAK1-HindIII-F/TaNAK1.1-N1-SalI-R, TaNAK1-HindIII-F/TaNAK1.2-N1-SalI-R, and TaNAK1-HindIII-F/TaNAK1.2-N2-SalI-R. The coding sequences of the carboxyl terminal that contain NB-ARC domains and all subsequent sequences) of the two protein isoforms were amplified with primer pair TaNAK1-C-SalI-F/TaNAK1-BamHI-R. These PCR products were separately digested by their restriction enzymes and subcloned into the p16318-GFP expression vector between the CaMV35S promoter and the *GFP* gene and fused with *GFP* in frame, generating transient expression vectors p16318-*TaNAK1.1*-*GFP*, p16318-*TaNAK1.2-GFP*, p16318-*TaNAK1.1^1-257^*-*GFP*, p16318-*TaNAK1.1^1-353^*-*GFP*, p16318-*TaNAK1.2^1-306^*-*GFP*, p16318-*TaNAK1.2^1-421^*-*GFP* and p16318-*TaNAK1.1/1.2^c-terminal^-*GFP, respectively. The resulted constructs and the control vector p16318-GFP were separately introduced into strain DH5α, and the endotoxin free plasmids of these vectors were extracted with GoldHi EndoFree Plasmid Maxi Kit (BOYAO Biotechnology, Shanghai, China). The p16318-*TaNAK1.1*-*GFP*, p16318-*TaNAK1.2-GFP*, p16318-*TaNAK1.1^1-257^*-*GFP*, p16318-*TaNAK1.1^1-353^*-*GFP*, p16318-*TaNAK1.2* ^1-306^-*GFP* p16318-*TaNAK1.2^1-421^*-*GFP*, and p16318-*TaNAK1.1/1.2^c-terminal^-*GFP as well as the control vector p16318-*GFP* were separately transformed or co-transformed with nuclear localization marker vector NLS-mCherry into wheat mesophyll protoplasts that were released from leaves of 6-10 days wheat seedlings by polyethylene glycol (PEG)-mediated transient expression system, followed by incubation in the dark at 25°C for 15h (Yoo et al., 2007). Moreover, the recombinant vectors were separately bombarded into onion epidermal cells *via* a gene gun system (Bio-Rad, United States), with p35S::GFP as a control, and the transformed cells were incubated on 1/2 Murashige and Skoog (MS) medium (Murashige and Skoog, 1962) in light or darkness for 36–48 h at 28°C. GFP, the subcellular localization of fusion proteins and mCherry in the transformed cell was observed with Leica inverted microscope DMi8 (Leica, Germany).

### Detection of the kinase activity of TaNAK1.1 and TaNAK1.2

In order to detect the kinase activity of TANAK.1 and TaNAK1.2, we first constructed prokaryotic expression vectors of the kinase domains of TaNAK1.1 and TaNAK1.2 using vector PGEX-6P-1 as a backbone. In detail, the coding sequences of the protein kinase domains in TaNAK1.1 and TaNAK1.2 were separately amplified with pMD19-T-TaNAK1.1 or pMD19-T-TaNAK1.2 as a template and primer pair containing restriction site, namely TaNAK1-kinase-EcoRI-F/TaNAK1.1-kinase-XhoI-R or TaNAK1-kinase-EcoRI-F/TaNAK1.2-kinase-SalI-R (**Supplementary Figure S1**). The PCR products were double digested with restriction enzymes, and subcloned into the prokaryotic expression vector pGEX-6P-1, respectively, and fused with the GST tag, generating prokaryotic expression vectors pGEX-6P-1-TaNAK1.1^44-257^ and pGEX-6P-1-TaNAK1.2^44-306^. These recombinant expression vectors were first confirmed by sequencing, and then the correct constructs were separately transferred into prokaryotic expression strain Rosetta (DE3) (Novagen, Malaysia). These transformants were used to express fusion protein GST-TaNAK1.1^44-257^ or GST-TaNAK1.2^44-306^ in large quantities at the condition of 0.5 mM IPTG, 28°C, 180 rpm, and the target proteins were purified with GST-Resin (Shang Hai Yue-ke Biotechnology CO.,LTD).

The kinase activity of the purified target proteins was determined by *in vitro* autophosphorylation activity. The buffer of kinase reaction *in vitro* includes 100 mM Tris, pH7.5, 10 mM MgCl_2_, 2 mM DTT (Dithiothreitol), and 0.5 mM ATP (Enders et al., 2017). CIAP (Takara Biotechnology (Dalian) Co. Ltd) was used for dephosphorylation reaction of target protein. The mixture of the purified protein and the kinase reaction buffer with or without CIAP was incubated at 25°C for 30 min, and 5×SDS PAGE buffer was used to stop the reactions. After the reactions being stopped, the target proteins were separated by 12.5% SDS–polyacrylamide gel electrophoresis (PAGE) at 15 mA for 1.5 h and transferred to nitrocellulose membranes. Anti-GST (Absin, Shanghai) was used to detect the target proteins with GST Tags. Phosphorylated target protein was detected using Phos-tag™ BTL-111 1mM (Lumiprobe, USA) according to a previous report (Kinoshita et al., 2012).

### Construction of *TaNAK1.1* and *TaNAK1.2* overexpression vectors and development of transgenic *Arabidopsis*

The coding sequences of *TaNAK1.1* and *TaNAK1.2* were amplified using primer pair TaNAK1-XbaI-F/TANAK1-BglII-R (**Supplementary Table S1**) and the pMD19-T-TaNAK1.1 or the pMD19-T-TaNAK1.2 as templates, respectively. The PCR products were double digested with restriction enzymes *XbaI* and *BglII*, and separately subcloned to pCAMBIA1304 (p1304) vector, between the CaMV35S promoter and the NOS terminator, generating plant expression vectors p1304-*TaNAK1.1* and p1304-*TaNAK1.2* (named as p35S::TaNAK1.1 and p35S::TaNAK1.2, respectively, for simplicity). The resulted expression vectors were first confirmed by sequencing and then separately transformed into *Agrobacterium tumefaciens* EHA105. The wild type plants of *Arabidopsis* (ecotype Columbia-0) were transformed with *Agrobacterium tumefaciens* EHA105 containing p35S::TaNAK1.1 or p35S::TaNAK1.2, respectively, by floral-dip method (Zhang et al., 2006). The mature seeds of the transformed *Arabidopsis* were cultured in the soil, and the plants were screened by spraying with 100 mg/L glufosinate (Basta) into the leaves of seedlings, and the positive transgenic plants that have resistance to Basta were transplanted and grown in phytotron as described above. The independent transgenic lines were continuously self-pollinated until homozygous lines were obtained.

### Quantitative real-time reverse transcriptase-polymerase chain reaction

*Arabidopsis* leaf samples of different genotypes were used to extract total RNA to determine the expression levels of the transgene *TaNAK1.1* or *TaNAK1.2*, and the flowering-related hub genes *AtFLC*, *AtFT*, *AtLFY*, and *AtAP1* by qRT-PCR. The cDNA products of transgenic lines and wild-type plants were normalised using *AtACTIN* (AT3G18780) as an internal reference gene, and three independent biological replicates were included. The PCR was conducted in triplicate for each RNA sample/primer combination. The primer sequences used were shown in **Supplementary Table S1**. The program of qRT-PCR was as follows: denaturation at 95°C for 30 s, followed by 40 cycles of 95°C for 5 s and 60°C for 30 s. The qRT-PCR was performed on CFX96 Touch Real-Time PCR Detection System (Bio-Rad, USA) using TB Green Premix Ex TaqII (TaKaRa Biotechnology (Dalian) Co. Ltd, Japan). For each PCR, the specificity of the amplification was validated and CT (the threshold cycle above background) values were calculated using Bio-Rad Cycler software, and PCR efficiency close to 100%.

The relative expression levels of the tested genes were analyzed according to the comparative 2^−ΔΔCT^ method reported previously (Livak and Schmittgen, 2001).

### Phenotype measurement of different genotypic *Arabidopsis*

The transition from vegetative to reproductive growth in *Arabidopsis* was expressed as bolting time (the time from sowing to the primary inflorescence reaching 1.0 cm in length). The number of rosette leaves was counted one week after bolting. Plant height and total number of branches per plant, including primary and secondary branches, were measured at maturity stage. After harvest, the total seed weight per plant and shoot biomass per plant were weighed using an electronic balance, and Harvest Index is calculated by dividing seed yield per plant by biomass per plant. More than 12 plants were tested for each independent transgenic line and wild-type. The seeds of different genotypic *Arabidopsis* were photographed with a stereomicroscope (SMZ25, Nikon) and Image J (https://imagej.nih.gov/ij/) was used for seed size statistics. More than 20 seeds were detected for each of the independent transgenic lines.

### Statistical analysis

Statistical analysis of the expression levels of target genes or phenotypic data was conducted using SPSS Statistics 26 (https://www.ibm.com/products/spss-statistics) and Duncan one-way ANOVA was performed. A significant difference was considered when *P*<0.05, and multiple comparisons or pairwise comparison (Student’s *t* test) were performed.

## Results

### Molecular characterization of *TaRK4B-1* in wheat

**Figure 1:**
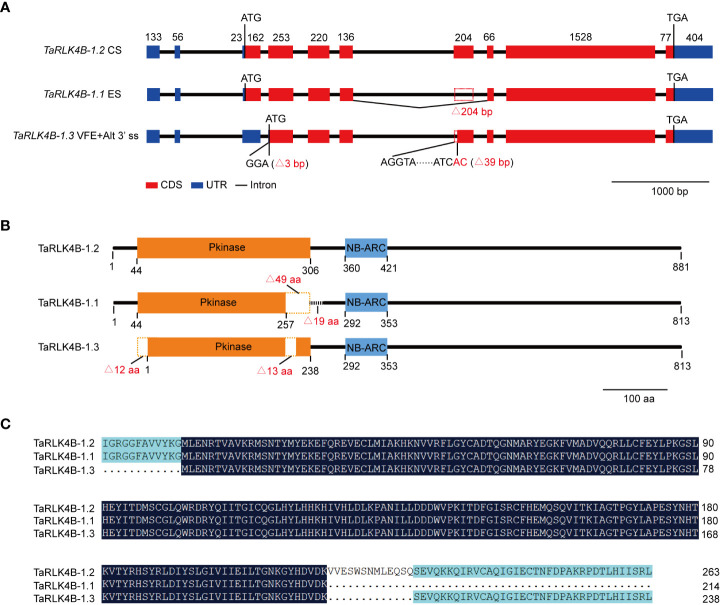
*TaRK4B-1* is subject to alternative splicing. **(A)** Gene structure of three alternative splicing variants *TaRK4B-1.1*, *TaRK4B-1.2* and *TaRK4B-1.3*. CS, constitutive splicing; ES, exon skipping; VFE, variable first exon; Alt 3’ ss, alternative 3’ splice site. **(B)** Schematic representation of the three TaRK4B-1 isoforms; Pkinase, protein kinase domain; NB-ARC, NB-ARC domain. **(C)** Amino acid sequence alignment of the protein kinase domains in the three TaRK4B-1 isoforms.

In order to uncover the differences among the three splicing variants of *TaRK4B-1*, we first analyzed the genomic structure of *TaRK4B-1* based on the IWGSC RefSeq v1.0 (Singh et al., 2018), and found that the full-length of *TaRK4B-1* locus encompasses a genomic DNA fragment of 5799 bp, including exons, introns, and 5’- and 3’- UTR (**Figure 1A**). We then compared the gene sequence with the three splicing variants and discovered that *TaRK4B-1.2* is a constitutive variant containing an ORF of 2646 bp, a 5’-UTR of 212 bp, and a 3’-UTR of 404 bp. *TaRK4B-1.1* arises from ES, leading to a deletion of 204 bp (772-975 bp) in the CDS, compared with *TaRK4B-1.2*. While *TaRK4B-1.3* is produced by VFE and Alt 3’ ss, resulting in deletion of 204 bp (1-165 and 772-810 bp) in the CDS, compared to *TaRK4B-1.2* (**Figure 1A**).

We further analyzed these splicing variants and their encoded proteins and found that *TaRK4B-1.2* encodes a protein composed of 881 amino acids (aa), that possesses two domains, namely the protein kinase domain (44-306 aa) and the NB-ARC domain (360-421 aa) (**Figure 1B**, **Supplementary Figure S1**). Compared with *TaRK4B-1.2*, *TaRK4B-1.1* misses a 204 bp fragment and the deletion does not result in frameshift mutations, thereby encoding a truncated protein isoform with a deletion of 49 aa (257-306 aa) in the protein kinase domain and subsequent 19 aa (307-325 aa), compared to the TaRK4B-1.2 (**Figures 1B, C**). Thus, the TaRK4B-1.1 contains a truncated protein kinase domain (44-257 aa) and the conserved NB-ARC domain (292-353 aa) (**Figures 1B, C**, **Supplementary Figure S1**). While *TaRK4B-1.3* produces a truncated protein isoform with a deletion of 56 aa (1-56 aa) in the amino terminal and a deletion of 13 aa (257-270 aa) within the protein kinase domain, compared with the TaRK4B-1.2; thus the TaRK4B-1.3 also possesses a truncated protein kinase domain (1-238 aa) and the conserved NB-ARC domain (292-353 aa) (**Figures 1B, C**, **Supplementary Figure S1**). Taken together, the three protein isoforms, TaRK4B-1.1, TaRK4B-1.2, and TaRK4B-1.3, all have the conserved NB-ARC domain and the protein kinase domain. However, the positions of the two domains and the length of the protein kinase domains are different among them, implying that they may have different three-dimensional structures and therefore have different biological functions. The three-dimensional structures of these three protein isoforms were predicated and the result exhibited their different three-dimensional structures (**Supplementary Figure S2**).

Considering the fact that *TaRK4B-1* gene encodes the proteins containing both the NB-ARC domain and the protein kinase domain in wheat, it was renamed as *NB-ARC domain containing protein kinase 1* (*TaNAK1*), correspondingly, the splicing variants *TaRK4B-1.1*, *TaRK4B-1.2*, and *TaRK4B-1.3* were renamed as *TaNAK1.1*, *TaNAK1.2*, and *TaNAK1.3*, respectively, in this study.

### Spatiotemporal expression patterns of the *TaNAK1* splicing variants in wheat

**Figure 2:**
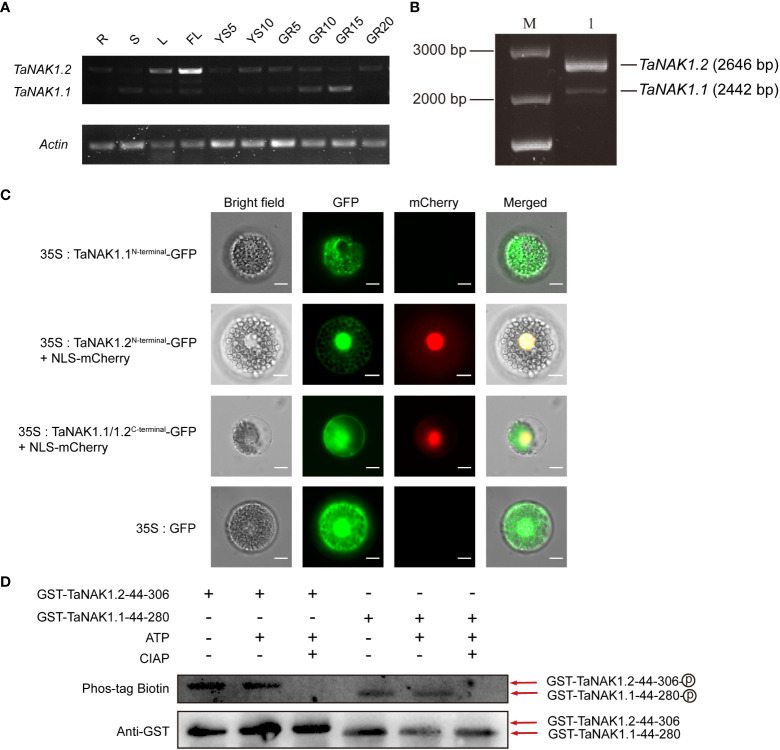
Expression patterns of *TaNAK1* splicing variants and subcellular localization and the autophosphorylation activities of two protein isoforms TaNAK1.1 and TaNAK1.2. **(A)** The spatiotemporal expression profiles of the splicing variants of *TaNAK1* in wheat detected by semi-quantitative reverse transcriptase-polymerase chain reaction with *Actin* (TraesCS1A01G020500) as a reference gene. *TaNAK1.1* and *TaNAK1.2*, two splicing variants of *TaNAK1*; *Actin*, a wheat housekeeping gene (TraesCS1A01G020500). R, root; S, stem; L, leaf; FL, flag leaf; YS5, the 5 cm long young earfrom wheat plants at booting stage; YS10, the 10 cm long spike from wheat plants at heading stage; GR5, GR10, GR15, and GR20, the grains 5, 10, 15, 20, and 25 days post-anthesis, respectively. **(B)** Amplification products of the *TaNAK1* transcripts obtained by RT-PCR; M, 250 bp DNA marker; 1, the products amplified with the gene-specific primer pair and the cDNA of wheat leaf as a template. **(C)** Subcellular localization of two protein isoforms TaNAK1.1 and TaNAK1.2 in wheat protoplasts. TaNAK1.1^N-terminal^, the N-terminals containing both the kinase domain and the NB-ARC domain (1-353 aa) of TaNAK1.1; TaNAK1.2^N-terminal^, the N-terminals containing both the kinase domain and the NB-ARC domain (1-421 aa) of TaNAK1.2; TaNAK1.1/1.2^C-terminal^, the C-terminal containing NB-ARC domain and all subsequent sequences (521aa at the carboxyl end) of TaNAK1.1/TaNAK1.2; NLS-mCherry, the Nuclear Localization Sequence short peptide fused with mCherry (red fluorescent protein). The vector control (35S:GFP) and fusion protein vectors (35S: TaNAK1.1^N-terminal^-GFP, 35S:TaNAK1.2^N-terminal^-GFP, and TaNAK1.1/1.2^C-terminal^-GFP) were each introduced into wheat protoplasts. GFP, the fusion proteins, and mCherry (red fluorescence) were observed with laser scanning confocal microscope; and Co-localization analysis of 35S:TaNAK1.2^N-terminal^-GFP or TaNAK1.1/1.2^C-terminal^-GFP (green) and NLS-mCherry (red) was observed by overlay. Bar = 10 μm. **(D)** The autophosphorylation activities of two protein isoforms TaNAK1.1 and TaNAK1.2 *in vitro*. Purified fusion proteins GST-TaNAK1.1^44-257^ and GST-TaNAK1.2^44-306^ were incubated independently with ATP and with or without CIAP in kinase reaction buffer, and their autophosphorylation activities were detected using Phos-tag Biotin (Wako, Japan). TaNAK1.1^44-257^and TaNAK1.2^44-306^ represents the kinase domains of TaNAK1.1 and TaNAK1.2, respectively. GST-TaNAK1.1^44-257^ and GST-TaNAK1.2^44-306^ indicate the protein kinase domains of TaNAK1.1 and TaNAK1.2 fused with GST, respectively. CIAP: Calf intestine alkaline phosphatase; Phos-tag Biotin: Biotinylated Phos-tag(Lumiprobe, USA); Anti-GST, GST antibody (Absin, Shanghai); : Phosphorylation.

Knowledge about the spatiotemporal expression of a gene might provide clues on where the gene functions. In order to explore the biological role of the three splicing variants of *TaNAK1* in wheat, we first analyzed the expression patterns of *TaNAK1* in different developmental stages and different tissues/organs of wheat cv. Xiaoyan 6 by conducting semi-qRT-PCR. The result showed that only two splicing variants *TaNAK1.1* and *TaNAK1.2* were detected, and they exhibit different expression patterns across ten tissues/organs, including R, S, L, FL, YS5, YS10, GR5, GR10, GR15, and GR20 (**Figure 2A**). *TaNAK1.1* is expressed in GR5, GR10, and GR15 to different degrees, with the highest expression level in GR15, while *TaNAK1.2* is mainly expressed in L and FL, with much higher expression level in FL than in L (**Figure 2A**). The splicing variant *TaNAK1.3* was not detected in any of the ten tissues tested (**Figure 2A**), implying its abundance too low to be measured. These results suggested that the two splicing variants *TaNAK1.1* and *TaNAK1.2* might play crucial and different biological roles in wheat growth and development. Therefore, *TaNAK1.1* and *TaNAK1.2* will be further investigated in subsequent studies.

### Subcellular localization of two protein isoforms TaNAK1.1 and TaNAK1.2 in wheat

To better understand the biological functions of *TaNAK1*, we first cloned and sequenced the complete coding sequences of *TaNAK1.1* and *TaNAK1.2* (**Figure 2B**). The results showed that the obtained sequence was identical to those described in IWGSC RefSeq v1.0. We then conducted subcellular localization of TaNAK1.1 and TaNAK1.2 by transient expression of fusion proteins TaNAK1.1-GFP and TaNAK1.2-GFP in wheat protoplasts and onion epidermal cells under the control of the CaMV35S promoter. Unfortunately, no green fluorescent signal was detected in transformed wheat protoplasts and onion epidermal cells, and we guessed that the fusion proteins were too large to be expressed transiently in wheat protoplast and onion epidermal cells. We next transiently expressed *GFP* fused with the N-terminals only containing the kinase domain (TaNAK1.1^1-257^ and TaNAK1.2^1-306^), the N-terminals containing both the kinase domain and the NB-ARC domain (TaNAK1.1^1-353^ and TaNAK1.2^1-421^), and the C-terminal containing NB-ARC domain and all subsequent sequences (521aa at the carboxyl end) of TaNAK1.1/TaNAK1.2 (named as TaNAK1.1/1.2^C-terminal^ for simplicity), respectively, in wheat protoplasts and onion epidermal cells, with the vector 35S::*GFP* being used as a positive control. The results exhibited that the green fluorescence of fusion proteins TaNAK1.1^1-257^-GFP (GFP fused with the N-terminal containing only the kinase domain of TaNAK1.1) and TaNAK1.1^1-353^-GFP (GFP fused with the N-terminal containing both the kinase domain and the NB-ARC domain of TaNAK1.1) was mainly observed in the cytoplasm of the transformed wheat protoplasts and onion epidermal cells (**Figure 2C** and **Supplementary Figure S3**). While the green fluorescence of fusion proteins TaNAK1.2^1-306^-GFP (GFP fused with the N-terminal containing only the kinase domain of TaNAK1.2) and TaNAK1.2^1-421^-GFP (GFP fused with the N-terminal containing both the kinase domain and the NB-ARC domain of TaNAK1.2) was observed in both the cytoplasm and the nucleus (**Figure 2C** and **Supplementary Figure S3**); And the green fluorescence of fusion protein TaNAK1.1/1.2^C-terminal^-GFP was observed in the cytoplasm and nucleus, which is similar to that of the control GFP (**Figure 2C** and **Supplementary Figure S3**). These suggested that TaNAK1.1 and TaNAK1.2 have different subcellular localization in wheat, and their subcellular localization was mainly determined by their respective N-terminal kinase domain sequences; TaNAK1.1 was located in cytoplasm, while TaNAK1.2 was located in both cytoplasm and nucleus.

### The kinase activity of two protein isoforms TaNAK1.1 and TaNAK1.2

Since the predicted kinase domains of the two protein isoforms TaNAK1.1 and TaNAK1.2 are different, we wondered whether they both have kinase activity. The kinase domains of TaNAK1.1 (44-257 aa) and TaNAK1.2 (44-306 aa) were expressed *in vitro*, and their autophosphorylation activities were detected using Phos-tag Biotin (Wako, Japan). The results showed that both protein isoforms could be autophosphorylated *in vitro* (**Figure 2D**), indicating that they both have kinase activity. However, whether there is a difference in kinase activity between these two protein isoforms needs further investigate.

### Ectopic expression of two splicing variants, *TaNAK1.1* and *TaNAK1.2*, confers opposite effects on the transition of *Arabidopsis* from vegetative growth to reproductive growth

**Figure 3:**
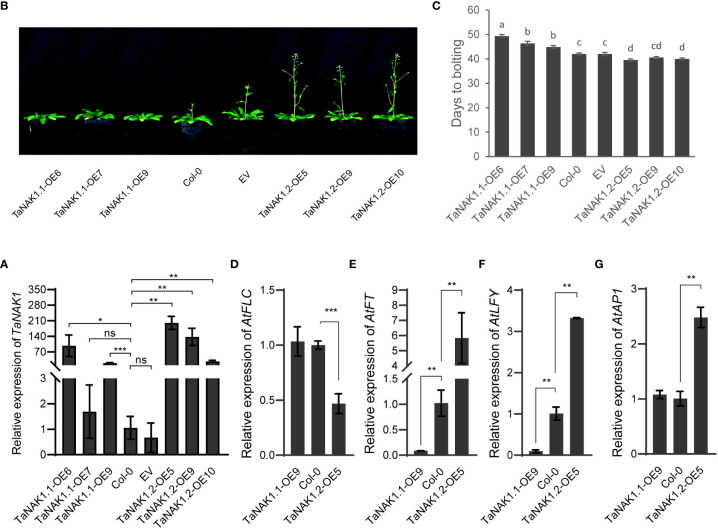
The effects of two *TaNAK1* splicing variants on the transition time from vegetative to reproductive growth of *Arabidopsis*. **(A)** The relative mRNA abundance of the *TaNAK1* splicing variants in transgenic lines and wild-type *Arabidopsis* plants determined by real-time quantitative RT-PCR (qRT–PCR). Values are given as the mean ± SE; three independent biological replicates included. * and ** indicate significant differences at *P < *0.05 and *P < *0.01 levels, respectively, compared with the Col-0 using Student’s *t* test; ns represent no significant differences. **(B)** The phenotypes of bolting in different genotypes of *Arabidopsis.* **(C)** Bolting time in different genotypes of *Arabidopsis*. Bolting time (the time from sowing to the primary inflorescence reaching 1.0 cm in length) represents the transition from vegetative to reproductive growth in *Arabidopsis*. **(D-G)** The relative expression levels of flowering-related genes *AtFLC*, *AtFT*, *AtLFY*, and *AtAP1* in different genotypes of *Arabidopsis* at 40 days post germination. Col-0: wild type *Arabidopsis*; EV: transgenic lines carrying empty vector p35S::Null; *TaNAK1.1*-OEs and *TaNAK1.2*-OEs: transgenic lines carrying expression vector p35S::*TaNAK1.1* and p35S::*TaNAK1.2*, respectively. The data indicate means ± SE (n ≥ 12), and significance analysis was performed using Duncan one-way Anova; The different lowercase letters above the error bars indicate the different significance level at *P* < 0.05; ** and *** indicate significant differences at *P < *0.01 and *P < *0.001 levels, respectively, compared with the Col-0 using Student’s *t* test. ns, no significant difference.

To explore the effects of the *TaNAK1* splicing variants on plant growth and development, expression vectors p35S::*TaNAK1.1* and p35S::*TaNAK1.2* as well as empty vector p35S::Null as a control were constructed and transferred into *Arabidopsis* ecotype Col-0, respectively, through *Agrobacterium*-mediated inflorescence dipping method (Zhang et al., 2006). More than 10, 10, and 5 independent transgenic *Arabidopsis* lines were developed for p35S::*TaNAK1.1*, p35S::*TaNAK1.2*, and p35S::Null construct, respectively. And finally, at least 5 independent transgenic homozygous lines for each genotype were obtained for further analysis. The mRNA levels of the *TaNAK1* splicing variants were first quantified in the transgenic lines carrying p35S::*TaNAK1.1* (*TaNAK1.1*-OEs) or p35S::*TaNAK1.2* (*TaNAK1.2*-OEs), and the transgenic lines carrying empty vector p35S::Null (EV) as well as wild-type *Arabidopsis* ecotype Col-0 (Col-0) by qRT-PCR. The results showed that *TaNAK1.1*-OEs and *TaNAK1.2*-OEs had significantly high abundance of the target genes, compared with Col-0 and EV, which had similar levels (**Figure 3A**), *TaNAK1.1*-OE6, 7, 9 and *TaNAK1.2*-OE5, 9, 10 being shown as representatives of *TaNAK1.1*-OEs and *TaNAK1.2*-OEs, respectively.

The phenotypes of *TaNAK1.1*-OEs, *TaNAK1.2*-OEs, EVs, and wild-type plants (Col-0) were then observed throughout the growth period of *Arabidopsis*. Firstly, very different bolting times were observed among these different genotypes of *Arabidopsis* (**Figure 3B**). Compared with Col-0, *TaNAK1.1*-OEs exhibited a delay in bolting time (3-7 days late), *TaNAK1.1*-OE6, *TaNAK1.1*-OE7, and *TaNAK1.1*-OE9 being shown as representatives (**Figures 3B, C**). In contrast, *TaNAK1.2*-OEs showed an advanced bolting time (2-3 days early), *TaNAK1.2*-OE5, *TaNAK1.2*-OE9, and *TaNAK1.2*-OE10 being shown as representatives (**Figures 3B, C**). While EV showed a similar bolting time with Col-0. Secondly, the very different number of rosette leaves was discovered among these different genotypes of *Arabidopsis* (**Supplementary Figure S3**). Compared with Col-0, *TaNAK1.1*-OEs had significantly increased number of rosette leaves at 7 days after bolting, but *TaNAK1.2*-OEs showed significantly decreased number of rosette leaves (**Supplementary Figures S4A, B**), which may be attributed to the length of their different vegetative growth periods. *TaNAK1.1*-OEs had an extended vegetative growth time due to late bolting, however *TaNAK1.2*-OEs had shortened vegetative growth time owing to early bolting. Taken together, *TaNAK1* splicing variants *TaNAK1.1* and *TaNAK1.2* have opposite effects on regulating flowering time in transgenic *Arabidopsis*, *TaNAK1.1* delays, while *TaNAK1.2* promotes the transition of *Arabidopsis* from vegetative growth to productive.

In *Arabidopsis*, flowering integration factor *FLOWERING LOCUS T* (*FT*) is involved in regulating the expression of transcription factors *FLEAY* (*LFY*) and *APETALA1* (*AP1*), the accumulation of appropriate amount of LFY and AP1 is the key to flower organ formation (Corbesier et al., 2007; Mathieu et al., 2007). But *FLOWERING LOCUS C* (*FLC*) as a negative regulator of flowering inhibits the transcription of *AtFT* (Helliwell et al., 2006; Krzymuski et al., 2015; Kinmonth-Schultz, 2021). To further elucidate the mechanism by which *TaNAK1.1* and *TaNAK1.2* affect flowering time, we investigated their effects on the above four hub genes regulating flowering, using *TaNAK1.1-*OE9 and *TaNAK1.2-*OE5 as representatives of *TaNAK1.1-*OEs and *TaNAK1.2-*OEs, respectively. The results exhibited that compared with Col-0, the expression levels of *AtFLC* and *AtAP1* were not significantly changed in *TaNAK1.1*-OE9, and the expression levels of *AtFT* and *AtLFY* were significantly decreased. However, the expression levels of *AtFLC* were significantly decreased in *TaNAK1.2*-OE5, and the expression levels of *AtFT*, *AtLFY*, and *AtAP1* were significantly increased, compared with Col-0 (**Figures 3D-G**). These indicated that overexpression of *TaNAK1.1* downregulated the expression of *AtFT* and *AtLFY* in transgenic *Arabidopsis*, while overexpression of *TaNAK1.2* downregulated the expression of *AtFLC* and thereby enhanced the expression of *AtFT*, *AtLFY* and *AtAP1* in transgenic *Arabidopsis*. These results are consistent with the phenotypes of *Arabidopsis* transition from vegetative growth to reproductive growth.

### Ectopic expression of the two splicing variants, *TaNAK1.1* and *TaNAK1.2*, has opposite effects on plant architecture and seed-size of *Arabidopsis*

**Figure 4:**
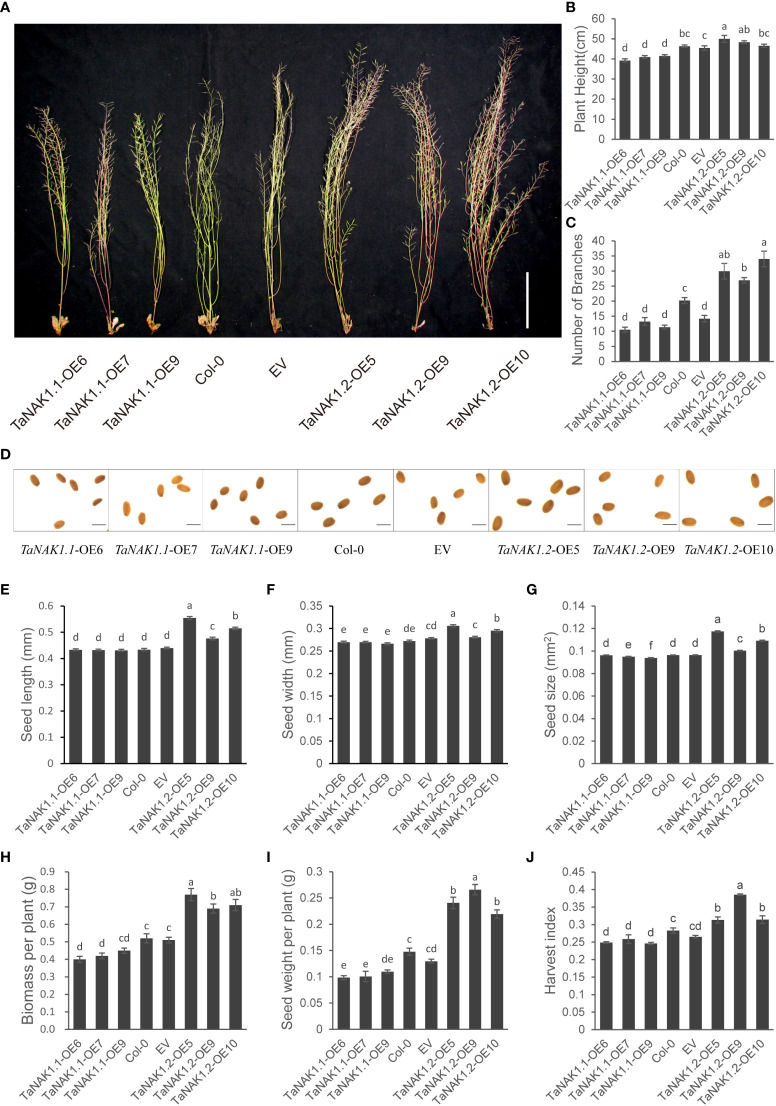
The effects of two *TaNAK1* splicing variants on plant architecture and seed yield related traits of *Arabidopsis*. **(A)** Images of different genotypes of *Arabidopsis* at maturity. Bar = 10 cm. **(B, C)** Plant height, the number of branches per plant of different genotypes of *Arabidopsis.* **(D)** Images of mature seeds from different genotypes of *Arabidopsis*. Bar = 0.5 mm. **(E-J)** Seed length, seed width, seed size, seed weight per plant, biomass per plant and harvest index of different genotypes of *Arabidopsis*. Col-0: wild type *Arabidopsis*; EV: transgenic lines carrying empty vector p35S::Null; *TaNAK1.1*-OE and *TaNAK1.2*-OE: transgenic lines carrying expression vector p35S::*TaNAK1.1* and p35S::*TaNAK1.2*, respectively. The data indicate means ± SE (n ≥ 12), and significance analysis was performed using Duncan one-way Anova; the different lowercase letters above the error bars indicate the different significance at *P* < 0.05 level.

The plant height and branch number of *Arabidopsis* at maturity were also investigated. The results showed that compared with the wild-type plants Col-0, *TaNAK1.1*-OEs exhibited decreased plant heights, while *TaNAK1.2*-OEs increased plant heights (**Figures 4A, B**), suggesting that *TaNAK1.1* and *TaNAK1.2* have opposite effects on plant height, *TaNAK1.1* and *TaNAK1.2* positively and negatively regulating plant height of transgenic *Arabidopsis*, respectively. Moreover, *TaNAK1.1*-OEs had the significantly reduced number of branches (11-13 branches), but *TaNAK1.2*-OEs showed the significantly increased number of branches (27-34 branches), compared with the wild-type plants Col-0 (20 branches) (**Figure 4C**); indicating that *TaNAK1.1* and *TaNAK1.2* have opposite effects on the number of branches of *Arabidopsis*.

We further examined the seed yield–related traits of *Arabidopsis*, including seed size, seed yield per plant, biomass per plant, and harvest coefficient. The results indicated that the seed length (0.4760-0.5543 mm) and width (0.2805 mm-0.3059 mm) of *TaNAK1.2*-OEs were significantly higher than those of the wild type (seed length: 0.4339 mm, seed width: 0.2722 mm) (**Figures 4D-F**). The detection of the seed sizes showed that compared with Col-0 (0.0963 mm^2^), *TaNAK1.1*-OEs had significantly decreased seed size (0.0938-0.0962 mm^2^), but *TaNAK1.2*-OEs increased seed size (0.1002-0.1174 mm^2^) (**Figures 4D, G**). Moreover, *TaNAK1.1*-OEs had lower biomass per plant (0.40-0.45 g) and seed yield per plant (0.098-0.100 g) than Col-0 (0.52 g, 0.15 g), however, *TaNAK1.2*-OEs possessed higher biomass per plant (0.69-0.77 g) and seed yield per plant (0.22-0.27 g) (**Figures 4H, I**). These suggest that *TaNAK1.1* and *TaNAK1.2* have opposite effects on the biomass and seed yield of transgenic *Arabidopsis*. Furthermore, *TaNAK1.1*-OEs had increased harvest index, but *TaNAK1.2*-OEs reduced harvest index, compared with the wild-type plants Col-0 (**Figure 4J**).

Taken together, ectopic expression of the two splicing variants, *TaNAK1.1* and *TaNAK1.2*, confers opposite effects on flowering time, plant architecture, and seed-yield related traits of *Arabidopsis*, *TaNAK1.1* negatively regulating the transition from vegetative growth to reproductive growth, plant height, branch number, biomass and seed yield per plant, while *TaNAK1.2* positively regulating these traits.

## Discussion

### A deletion of 49 amino acid residues in the protein kinase domains may be important to the functional differences between *TaNAK1.1* and *TaNAK1.2*

The existence of AS in plants indicates the specialization of gene function in biological evolution, which is a universal phenomenon in organisms. The expression patterns of different transcripts produced by the same gene, the functional domain positions of the proteins encoded by these transcripts, and the biological roles of these proteins may be different (Swarup et al., 2016; Qin et al., 2017; Huang et al., 2019). For examples, in rice, 18 nitrate and di/tripeptide transporter (NPF) genes undergo alternative splicing, producing 36 different transcripts. Of these, *OsNPF7.1* and *OsNPF7.4* exhibited opposite expression patterns in axillary buds, the expression levels of the two alternative splicing variants determine the growth of axillary buds, especially for the second bud, and subsequently affect the tillering number of rice (Huang et al., 2019). In *Musa nana* Lour., *MYB16* gene generates two alternative splicing variants, *MaMYB16L* and *MaMYB16S*, which play opposite functions in the regulation of fruit ripening. *MaMYB16L* is a transcription inhibitor that directly downregulates the expression of *MaDREB2* to inhibit fruit ripening; *MaMYB16S* loses its ability to bind to DNA but can competitively bind with *MaMYB16L*, forming inactive dimers that can promote fruit ripening (Jiang et al., 2021). In wheat, the Gγ subunit gene *TaGS3* undergoes AS and produces five splicing variants, namely, the constitutive *TaGS3.1*, the truncated *TaGS3.2–3.4* with the C-terminal Cys-rich region missing, and the truncated TaGS3.5 that contains disrupted OSR domain; and the five splicing variants exhibited expression divergence in wheat polyploidization, and differential function in controlling grain weight and size (Ren et al., 2021).

In this study, the two transcripts of *TaNAK1*, *TaNAK1.1* and *TaNAK1.2*, were experimentally identified. Constitutive *TaNAK1.2* encodes a protein composed of 881 amino acids that contains a protein kinase domain (44-306 aa) and a NB-ARC domain (360-421 aa), while *TaNAK1.1* misses an exon with a length of 204 bp, compared with *TaRK4B-1.2*, thereby encoding a truncated protein isoform with a deletion of 49 aa (257-306 aa) in the kinase domain and subsequent 19 aa (307-325 aa), compared with TaRK4B-1.2 (**Figures 1B, C**); The positions of the two functional domains and the length of the protein kinase domains between the two protein isoforms are different, which makes them to have different three-dimensional structures (**Supplementary Figure S2**) and therefore have different biological functions. *In vitro* autophosphorylation activity analysis of using Phos-tag Biotin (Wako, Japan) showed that both TaNAK1.1 and TaNAK1.2 have protein kinase activity (**Figure 2D**). Furthermore, the subcellular localization of the protein isoforms was conducted in this stuby. Because the full-length of the two protein isoforms fused with GFP was too large to be expressed in wheat protoplasts and in onion epidermal cells, we tried to construct different length of TaNAK1.1 and TaNAK1.2 into expression vector to solve this problem. The result exhibited that the fusion proteins TaNAK1.1^1-257^-GFP and TaNAK1.1^1-353^-GFP was mainly observed in the cytoplasm of the transformed wheat protoplasts and onion epidermal cells, while the proteins TaNAK1.2^1-306^-GFP and TaNAK1.2^1-421^-GFP was observed in the cytoplasm and nucleus; And the fusion protein TaNAK1.1/1.2 ^C-terminal^-GFP was observed in the cytoplasm and nucleus, which is similar as that of the control GFP (**Figure 2C** and **Supplementary Figure S3**). These suggested that TaNAK1.1 and TaNAK1.2 have different subcellular localization in wheat, TaNAK1.1 mainly in the cytoplasm, while TaNAK1.2 in both the nucleus and cytoplasm; And their subcellular localization was mainly determined by their respective N-terminal kinase domain sequences. These data suggested that these two protein isoforms might have different biological roles. Ectopic expression of the two splicing variants, *TaNAK1.1* and *TaNAK1.2*, has opposite effects on flowering time, plant architecture, and seed-yield related traits of *Arabidopsis*, *TaNAK1.1* negatively regulates the transition time from vegetative growth to reproductive growth, plant height, branching number, biomass and seed yield per plant, while *TaNAK1.2* positively regulates these traits (**Figures 3** and **Figure 4**). Based on previous relevant reports, we preliminarily suppose that there may be two main reasons for this difference. One is that the deletion of a 204-bp exon leads to a 49-aa difference in the kinase domains and a subsequent 19-aa difference between TaNAK1.1 and TaNAK1.2, which results in a difference in subcellular localization of the two protein isoforms. This may be the key to the phenotypic differences. Another is that the differences in the positions of two functional domains and the length of the protein kinase domains of TaNAK1.1 and TaNAK1.2 may result in their different three-dimensional structures and therefore binding different substrates. This might be one of the possible reasons for the phenotypic differences. However, this needs further demonstrate.

### *TaNAK1.1* and *TaNAK1.2* differentially affecting *Arabidopsis* transition from vegetative growth to reproductive growth may be attributed to the differential expression of *AtFT* and *AtLFY* genes

Flowering at the right moment is crucial for the reproductive success of flowering plants. The regulation of flowering time in model plant species *Arabidopsis* has been extensively studied. A great deal of information is available about the factors involved and how they interact genetically. For examples, in *Arabidopsis* leaves, *SHORT VEGETATIVE PHASE* (*SVP*) and *FLC* repress *FT* transcription (Helliwell et al., 2006; Jang et al., 2010). *FT* is produced in leaves and moves to shoot apical meristem (SAM) to interact with FD to form complex that activates *SUPPRESSOR OF OVEREXPRESSION OF CONSTANS1* (*SOC1*) and *AP1* expression on SAM (Abe et al., 2005; Yoo et al., 2005; Corbesier et al., 2007; Mathieu et al., 2007). In addition, *LFY* plays a key role in the integration of flowering signals parallel to *FT* to activate floral meristem identity genes such as *AP1* triggering flowering (William et al., 2004; Abe et al., 2005). And *FT* can activate *LFY* expression through the transcription factor SOC1 (Yoo et al., 2005; Lee et al., 2008). A core regulatory network of eight integrated flowering genes has been established (Jaeger et al., 2013; Leal Valentim et al., 2015). In general, the floral pathway integrator genes *FT* and *LFY* play key roles in higher plants. Environmental and endogenous floral promoting signals stimulate the expression of floral pathway integrator genes *FT* and *LFY*. Elevated levels of *FT* and *LFY* proteins stimulate the expression of floral meristem identity genes, such as *AP1*, leading to flowering.

In the current study, in order to elucidate the mechanisms by which *TaNAK1.1* and *TaNAK1.2* differentially affect the transition from vegetative growth to reproductive growth in *Arabidopsis*, we investigated their effects on the four hub genes regulating flowering, including *AtFLC*, *AtFT*, *AtLFY*, and *AtAP1*, using *TaNAK1.1-*OE9 and *TaNAK1.2-*OE5 as the representatives of *TaNAK1.1-*OEs and *TaNAK1.2-*OEs, respectively. Our results demonstrated that ectopic overexpression of *TaNAK1.1* and *TaNAK1.2* had opposite effects on the expression of *AtFT* and *AtLFY* in transgenic lines, *TaNAK1.2* upregulating while *TaNAK1.1* downregulating the expression of *AtFT* and *AtLFY* (**Figures 3E, E**). These are consistent with the phenotypes of advanced flowering in *TaNAK1.2-*OEs and delayed flowering in *TaNAK1.1-*OEs, indicating that the main reason for *TaNAK1.1* and *TaNAK1.2* differentially affecting *Arabidopsis* transition from vegetative growth to reproductive growth may be that they differentially regulate the expression of *AtFT* and *AtLFY* genes in *Arabidopsis*.

### The differential expression patterns and subcellular localization of TaNAK1.1 and TaNAK1.2 in wheat may lead to different roles in regulating plant architecture and grain-yield

Plant architecture is defined as the three-dimensional organization of the whole plant. Tillering/branching and plant height are crucial traits that determine plant architecture and grain yield of cereal crops (Khush, 2003; Wang and Li, 2008; Alqudah et al., 2016; Jian et al., 2022). Plant architecture is one of the most important factors affecting grain yield. Rice researchers have proposed a model of ideal plant architecture (IPA) that should have a low tiller number; a high number of productive tillers (9-10); 200–250 grains per panicle; erect leaves; and vigorous and deep root systems (Wang and Li, 2008). A striking example is the Green Revolution, the development of semi-dwarf wheat and rice varieties that have greatly increased crop production since 1960. Great advances have been made in understanding the molecular mechanisms underlying plant structure formation over the past decade (Lee et al., 2008; Wang et al., 2018). Numerous studies have revealed the genetic basis of rice tillering using natural or artificially induced mutants, transgenic plants as well as specific loss-of-function mutants generated by genome editing, uncovering the functions of vital genes such as *MOC1 (MONOCULM1)* that encodes a transcriptional regulator of axillary meristem formation and can be used to enhance the number of tillers (Li et al., 2003), and *CCA1* (*CIRCADIAN CLOCK ASSOCIATED 1*) and *PRR1* (*PSEUDORESPONSE REGULATOR 1*) that have opposite effects on tiller numbers: overexpressing *CCA1* or knocking-out *PRR1* reduces tiller number, whereas knocking-out *CCA1* or overexpressing *PRR1* exhibits the opposite effect (Wang et al., 2020). In addition, *CCA1* has also been demonstrated to regulate *IPA1/SPL14*, a transcription factor of the SQUAMOSA promoter binding protein family, which has been implicated in the ideal plant architecture of rice (Jiao et al., 2010; Miura et al., 2010). In barley, alternative splicing of a gene *HORVU2Hr1G098820*, which encodes a trypsin family protein, produces a non-functional protein that results in excessive tillering and semi-dwarfing, leading to increased yield (Hua et al., 2020). In rice, two splicing variants of *OsNPF7, OsNPF7.1* and *OsNPF7.4*, exhibited opposite expression patterns in axillary buds under different nitrogen concentrations, the expression levels of *OsNPF7.1* and *OsNPF7.4* determining the axillary bud outgrowth and subsequently influencing the tiller number, therefore, differentially regulating tiller and grain yield (Huang et al., 2019). In the present study, the two splicing variants of *TaNAK1*, *TaNAK1.1* and *TaNAK1.2*, exhibit differential expression patterns during wheat growth and development, *TaNAK1.1* mainly is expressed in developmental grains, whereas *TaNAK1.2* in L and FL (**Figure 2A**), this suggesting that *TaNAK1.1* and *TaNAK1.2* might play crucial but different biological roles in wheat growth and development. The two protein isoforms, TaNAK1.1 and TaNAK1.2, exhibit different subcellular localization in wheat, TaNAK1.1 mainly in the cytoplasm, while TaNAK1.2 in the cytoplasm and nucleus (**Figure 2C**). Further analysis of the kinase activity of TaNAK1.1 and TaNAK1.2 demonstrated that they both have kinase activity *in vitro* (**Figure 2D**). However, whether they may bind different substrates needs to be further studied. In order to rapidly explore the effects of *TaNAK1.1* and *TaNAK1.2* on plant architecture and seed yield, transgenic *Arabidopsis* lines overexpressing the two splicing variants were developed and investigated. Our research data indicated that the two splicing variants have opposite effects on plant height and branch number of *Arabidopsis, TaNAK1.2* positively regulates plant height and the number of branching, while *TaNAK1.1* negatively regulates these traits (**Figures 4A-C**). In addition, *TaNAK1.1* and *TaNAK1.2* have opposite effects on *Arabidopsis* seed-size, biomass and seed yield per plant as well as harvest index, *TaNAK1.2* increases seed size, biomass and seed yield per plant, and harvest index, while *TaNAK1.1* decreases these traits (**Figures 4D-J**). In short, *TaNAK1.1* and *TaNAK1.2* have different effects on plant architecture and seed-yield related traits of *Arabidopsis.* Combining with differential expression patterns and subcellular localization of TaNAK1.1 and TaNAK1.2 in wheat, we speculate that *TaNAK1.1* and *TaNAK1.2* may have different functions in wheat growth and development. However, whether *TaNAK1.1* and *TaNAK1.2* play different roles in regulating wheat plant architecture and grain yield remains to be further verified by using transgenic wheat and loss-of-function mutants of *TaNAK1* generated by CRISPR/Cas9 genome editing, and this research work is in progress.

## Data availability statement

The original contributions presented in the study are included in the article/**Supplementary Material**. Further inquiries can be directed to the corresponding authors.

## Author contributions

HXZ and MM conceived and designed the experiments. BW and XZ conducted the experiments and did data analysis. KH, HYZ and SZ did investigation on the phenotypic traits of *Arabidopsis*. XL provided with helpful discussion. BW and XZ wrote the draft. HXZ and MM revised the manuscript. All authors contributed to the article and approved the submitted version.

## Funding

This study was financially supported by the Natural Science Foundation of China (32072003 and 32072059), and the Key Research and Development Program of Shaanxi Province (2021NY-079).

## Acknowledgment

We give thanks to Prof. Rudi Appels, Honorary Professor, University of Melbourne, for reading and improving the draft of this manuscript.

## Conflict of interest

The authors declare that the research was conducted in the absence of any commercial or financial relationships that could be construed as a potential conflict of interest.

The reviewer HM declared a shared affiliation with the authors to the handling editor at the time of review.

## Publisher’s note

All claims expressed in this article are solely those of the authors and do not necessarily represent those of their affiliated organizations, or those of the publisher, the editors and the reviewers. Any product that may be evaluated in this article, or claim that may be made by its manufacturer, is not guaranteed or endorsed by the publisher.
