# Supplementary material for: Two alternative splicing variants of a wheat gene TaNAK1, TaNAK1.1 and TaNAK1.2, differentially regulate flowering time and plant architecture leading to differences in seed yield of transgenic Arabidopsis
Source: Front Plant Sci. 2022 Dec 1;13:1014176. doi: 10.3389/fpls.2022.1014176 (PMC9751850; doi:10.3389/fpls.2022.1014176)
Supplement: Supplementary file 1 [file DataSheet_1.pdf]

Supplementary materials

Two alternative splicing variants of a wheat gene *TaNAK1*, *TaNAK1.1* and *TaNAK1.2*, have the opposite roles in flowering time and plant architecture leading to differences in seed yield of transgenic *Arabidopsis*

Baowei Wu*,Xiaoyu Zhang*, Kunzhi Hu, Haoyuan Zheng, Siyu Zhang, Xiangli Liu, Meng Ma#, Huixian Zhao#

Correspondence: Corresponding author: Huixian Zhao hxzhao212@nwafu.edu.cn, Meng Ma mengma5@nwafu.edu.cn.

1. Supplementary Tables

Table S1. List of the primers used in this study.

Primer name	Primers sequence (5'→3')	Use
TaNAK1-F	ATGGATCACCAATCTGTTATTACACCA	cDNA cloning
TaNAK1-R	TACTTTCTTGCGATGCCAAAAGC	
TaNAK1-XbaI-F	gagaacacgggggactctagaATGGATCACCAATCTGTTATTACACC	construction of p1304-*TaNAK1*
TaNAK1-BglII-R	tctcctttactagtcagatctacTACTTTCTTGCGATGCCAAAAG	
TaNAK1-SalI-F	cttgcatgcctgcaggtcgacATGGATCACCAATCTGTTATTACACC	Construction of p16318-TaNAK1.1-GFP and p16318-*TaNAK1.2*-GFP
TaNAK1-BamHI-R	gcccttgctcaccatggatccTACTTTCTTGCGATGCCAAAAG	
TaNAK1- HindIII-F	cccaagcttATGGATCACCAATCTGTTATTACACC	Construction of p16318-TaNAK1.1$^{1-257}$-GFP and p16318-TaNAK1.1$^{1-353}$
TaNAK1.1-N1-SalI-R	acgcGTCGACCTCCTCTGAGCTTGATTCTCTTGG	
TaNAK1.1-N2-SalI-R	acgcGTCGACACAACTGATCTTGACAAACCCTTG	

TaNAK1.2-N1- SalI-R	acgcGTCGACGAGTCTACTAATTATATGTAGTGTATCTGGTCTC	Construction of p16318-*TaNAK1.2*$^{1\text{-}306}$-GFP and p16318-*TaNAK1.2*$^{1\text{-}421}$
TaNAK1.2-N2- SalI-R	tcctctagagatatcgtcgacTACTTTCTTGCGATGCCAAAAG	
TaNAK1-C- SalI-F	tggagaggacagcccaagcttAGTCTTGATCCAGACATCAAGCG	Construction of p16318-*TaNAK1.1/1.2*$^{c\text{-}terminal}$ -GFP
TaNAK1-Kinase-EcoRI-F	ccgGAATTCATGGATCACCAATCTGTTATTA	pGEX-6P-1-*TaNAK1.1*$^{44\text{-}257}$ construction、pGEX-6P-1-*TaNAK1.2* $^{44\text{-}306}$ construction
TaNAK1.1-Kinase-XhoI-R	ccgctcgagTCAGAACATGTCAAGGTT	
TaNAK1.2-Kinase-SalI-R	acgcGTCGACGAGTCTACTAATTATATGTAGTGTATCTG	
TaNAK1-semiq PCR-F	TATTGTTGGATGATGATTGGGTAC	Semi–quantitative RT-PCR
TaNAK1-semiq PCR-R	GATTCTCTTGGCGTGTTAGGTG	
TaACTIN-semiqPCR-F	AAATCTGGCATCACACTTTCTAC	Semi–quantitative RT-PCR
TaACTIN-semiqPCR-R	GTCTCAAACATAATCTGGGTCATC	
TaNAK1-qPCR-F	TGCGCCACATAAATTCCG	qRT-PCR
TaNAK1-qPCR-R	AGTTCGCTGAGCCGTTCC	
AtACTIN-qPCR-F	AAATCTGGCATCACACTTTCTAC	qRT-PCR
AtACTIN-qPCR-R	GTCTCAAACATAATCTGGGTCATC	
AtFLC-F	CGATGCTCTTGTTCAACTGG	qRT-PCR
AtFLC-R	CTTCTGCTCCCACATGATGA	
AtFT-F	GCTTGGATCTAAGGCCTTCTCA	qRT-PCR
AtFT-R	TGGGACTTGGATTTTCGTAACA	
AtLFY-F	ACGCCGTCATTTGCTACTCT	qRT-PCR
AtLFY-R	CTTTCTCCGTCTCTGCTGCT	

AtAP1-F	GAAGGCCATACAGGAGCAAA	qRT-PCR
AtAP1-R	ACTGCTCCTGTTGAGCCCTA	

Note: F, forward primer, R, reverse primer

2. Supplementary Figures

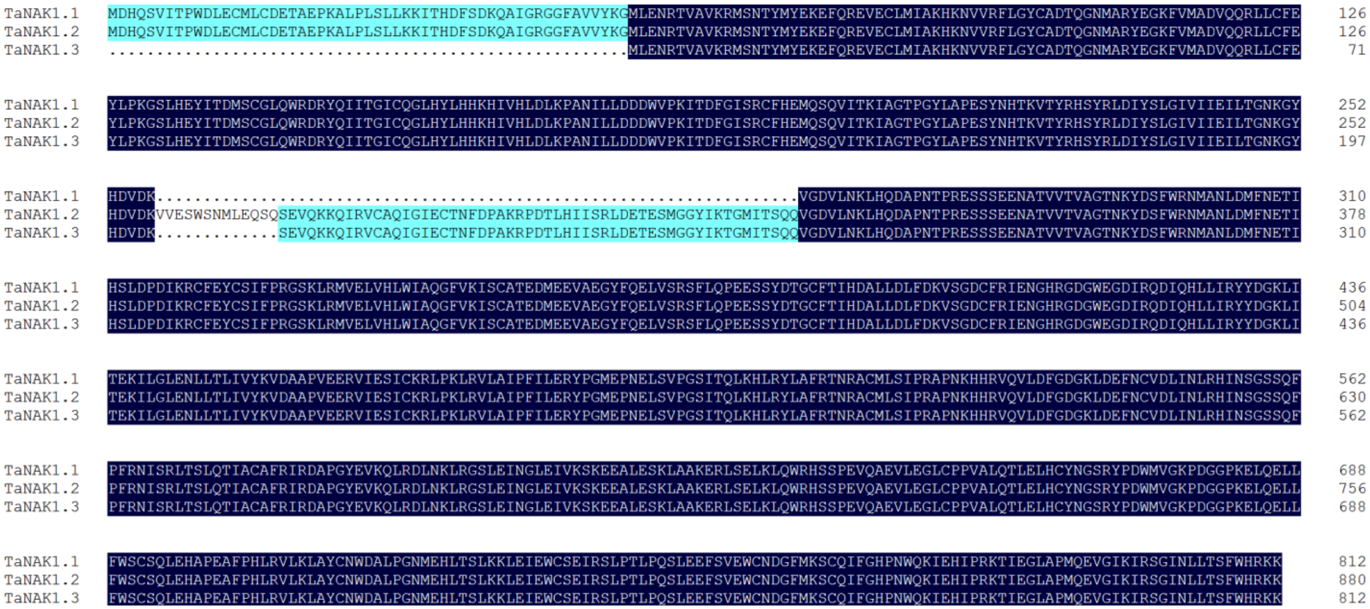

Figure S1. Alignment of full-length amino acid sequence of three protein isoforms of *TaNAK1*gene in wheat.

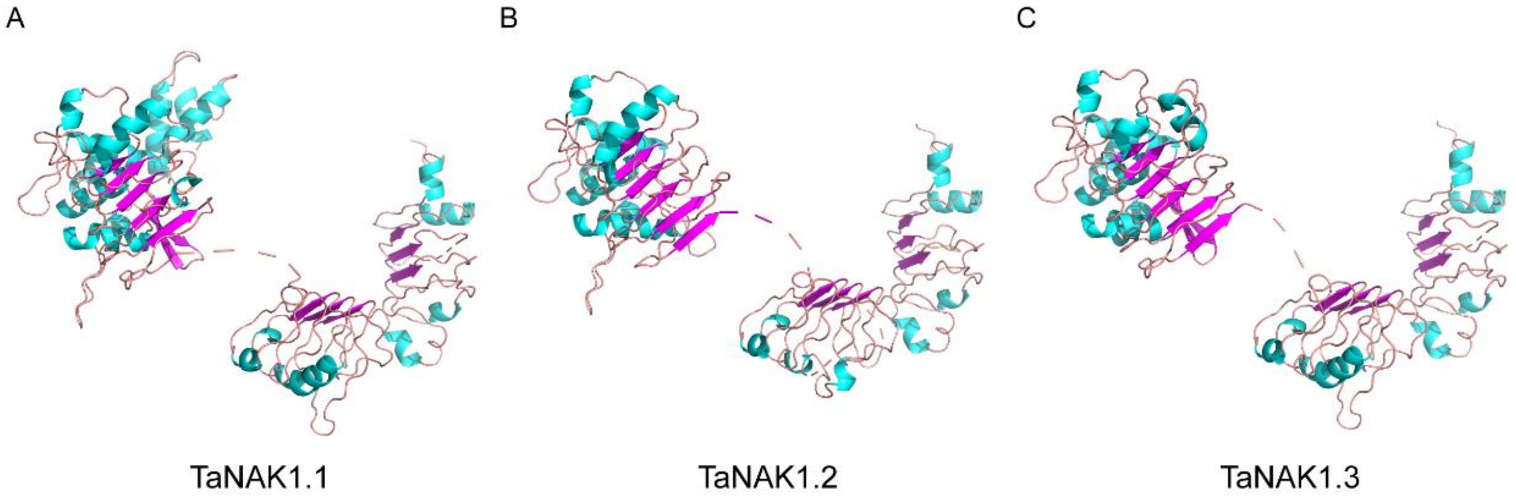

Figure S2. TaNAK1.1, TaNAK1.2 and TaNAK1.3 form different 3D structures. (A) Cartoon model of TaNAK1.1 done using the Phyre2 based on plant nlr rpp1 tetramer in complex with atr1 (PDB: 7crc, Fold library id: c7crcB) with 18% of identity and 100.0% confidence. (B) Cartoon model of TaNAK1.1 done using the Phyre2 based on plant nlr rpp1 tetramer in complex with atr1 (PDB: 7crc, Fold library id: c7crcB) with 17% of identity and 100.0% confidence. (C) Cartoon model of the TaNAK1.1 done using the Phyre2 based on plant nlr rpp1 tetramer in complex with atr1 (PDB: 7crc, Fold library id: c7crcB) with 20% of identity and 100.0% confidence.
Cyan indicates helix, magenta indicates beta sheet, orange indicates loop. TaNAK1.1 contains 13 α helices and 19 β sheets, TaNAK1.2 has 12 α helices and 16 β sheets, while TaNAK1.3 contains 14 α helices and 16 β sheets.

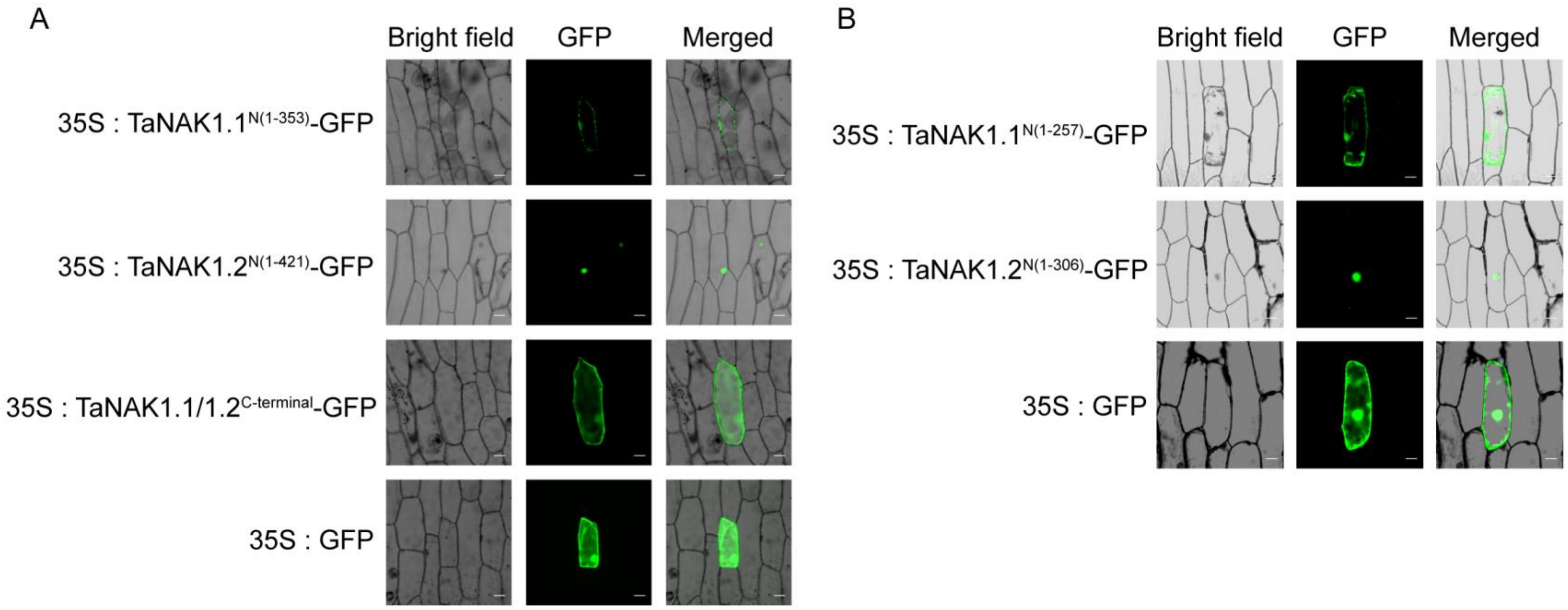

Figure S3 Subcellular localization of two protein isoforms TaNAK1.1 and TaNAK1.2 in onion epidermal cells.

(A) Subcellular localization of two protein isoforms by transiently expressing their N-terminals containing both the kinase domain and the NB-ARC domain (TaNAK1.1$^{N(1\text{-}353)}$ and TaNAK1.2$^{N(1\text{-}421)}$) separately fused with GFP in onion epidermal cells. (B) Subcellular localization of two protein isoforms by transiently expressing their N-terminals only containing the kinase domain (TaNAK1.1$^{N(1\text{-}257)}$ and TaNAK1.2$^{N(1\text{-}306)}$) separately fused with GFP in onion epidermal cells. The vector control (35S:GFP) and fusion protein vectors (35S: TaNAK1.1 $^{N(1\text{-}353)}$-GFP, 35S:TaNAK1.2$^{N(1\text{-}421)}$-GFP, 35S:TaNAK1.1/1.2 $^{C\text{-}terminal}$-GFP, TaNAK1.1$^{N(1\text{-}257)}$, and TaNAK1.2$^{N(1\text{-}306)}$) were each introduced into onion epidermal cells, and GFP and fusion proteins were monitored laser scanning confocal microscope. TaNAK1.1/1.2 $^{C\text{-}terminal}$, the C-terminal containing NB-ARC domain and all subsequent sequences (521aa at the carboxyl end) of TaNAK1.1/TaNAK1.2. Bar= 50 μm.

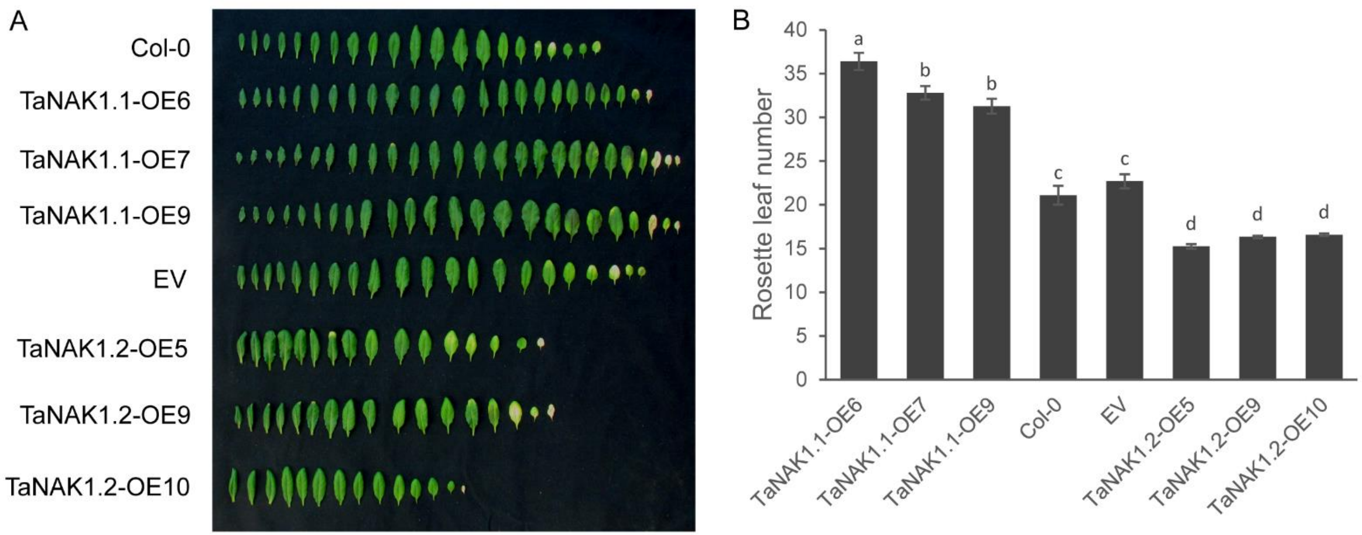

Figure S4. The phenotypes of rosette leaf from different genotypic *Arabidopsis*. (A) Photos of rosette leaves of different genotypic *Arabidopsis*. (B) Statistics of the number of rosettes leaves from different genotypic *Arabidopsis*. Col-0: wild type *Arabidopsis*; EV: transgenic lines carrying empty vector p35S::Null; *TaNAK1.1*-OE and *TaNAK1.2*-OE: transgenic lines carrying expression vector p35S::*TaNAK1.1* and p35S::*TaNAK1.2*, respectively. The data indicate means ± SE ($n \geq 12$), and significance analysis was performed using Duncan one-way Anova. The different lowercase letters above the error bars indicate the different significance level at $P < 0.05$.
